# Training in Surgical Efficiency

**Published:** 1916-02-26

**Authors:** 


### TRAINING IN SURGICAL EFFICIENCY.

### An Original College of Surgery.

In the February issue of the *Practitioner*, Mr. J. H. Watson, F.R.C.S., of Burnley, pleads for better facilities and training in surgery for young practitioners, especially for those whose practice is destined to be in provincial towns away from large schools of medicine. He holds up for emulation in this country the work done in this direction by the American College of Surgeons. This comparatively new organisation is quite different in scope and aim from our own Royal Colleges of Surgeons in England, Scotland, and Ireland; it is a society, rather than a teaching or an examining institute, and it now numbers some 3,400 Fellows in Canada and the United States. In view of the fact that it has quite recently raised an endowment fund from among its own Fellows of more than £100,000, and of its unique character, the indications of its purpose afforded by a paper contributed to the *American Journal of Obstetrics** by its director, Dr. J. G. Bowman, are of great interest to surgeons in this country.

### The American College of Surgeons.

The College, he states, will systematically seek information as to the character and attainments of such senior students and young practitioners who register their names as desirous of taking up surgery and of joining the College, and the information thus obtained will become the basis of admission to the Fellowship. Moreover, the "Regents"—apparently the managing committee—of the College will insist on the proper keeping of case histories, and will also encourage high ideals in medical practice. "Inasmuch as proper training in surgery is inseparably involved with the conduct and efficiency of hospitals, the College will seek accurate data on all matters which relate to hospitals"; a fairly large order this, as we can testify of our own knowledge. To show in what intricate matters the College proposes to interest itself, it may be enough to state that it will investigate institutional equipment for medical diagnosis (laboratories, *x*-rays, etc.); the taking and keeping of proper case records; the management and curricula of the nurse-training schools; the question of adequate specialisation in hospitals. In the last but one of these departments, it will be noticed, the American College is taking to itself functions similar to those which Mr. Arthur Stanley would allocate to his suggested College of Nursing in this country. We confess we think that nursing matters would be better left to a nursing authority than to a surgical one; and the physicians may have a word to say if the American College of Surgeons tries to push its influence too far in this direction.

But there are even larger problems which the College intends to tackle. It will ask the faculties of medical schools to consider the advisability of conferring a supplementary degree of proficiency in general surgery and in the various specialities of surgery. This programme seems on the face of it to conflict with the College's effort to constitute its own Fellowship the passport to public confidence in a surgeon. In this country so many universities already grant degrees in surgery that we doubt if any suggestion of this kind would be in the least feasible. To show the lines on which the American Fellowship is to be granted, the following list of conditions to be observed by the candidate after qualifying is instructive. The practitioner must serve at least one year as a house officer, preferably in a hospital with a varied service; must serve at least three years as second assistant or one year as first assistant to a surgeon of recognised ability with an adequate hospital clinic; and must report at least 100 cases in which he has thus acted as assistant. He must also produce evidence that he has visited other surgical clinics besides those at which he has been in office. He must give a list of his own publications, and show that he has done some work for the advancement of the general cause of surgery; and, finally, he must report fifty consecutive major operations done by himself.

It will be seen that this is an attempt to break away from the traditional examination system and to obtain some surer guarantee of professional efficiency. Mr. Watson approves of the principles thus outlined, and holds that they "might be copied here with advantage to all concerned." Admittedly the examination system has many defects; and it is very possible that this alternative to it might produce much better results. No system is infallible, however, and it is quite clear that the scheme of the American College of Surgeons cannot guarantee that all its Fellows will be first-rate surgeons, though it may more nearly reach that ideal than, say, the Fellowship examinations of the Royal Colleges in Great Britain do. There are obvious opportunities in the American scheme for what in the Services is known as "eyewash"; and we cannot help suspecting that if the American College gets its schemes accepted as universally as its director hopes, a good deal of that emollient liquor will be applied to the eyes of its Regents. In America there are many past-masters in this branch of ophthalmic practice, which may possibly make the Regents wary; but we have our doubts about it.

### A Surgical Creed.

Again, the fellowship pledge is worth quoting, though we cannot help thinking that some of the evils it seeks to guard against are not to be stopped by any system of pledges. At any rate, the sentiments expressed in it are unexceptionable. The pledge runs thus: "In particular, I pledge myself to pursue the practice of surgery with thorough self-restraint, and to place the welfare of my patients before all else; to advance constantly in knowledge by the study of surgical litera-

* January 1916, p. 161.

ture, the instruction of eminent teachers, interchange of opinion amongst my associates, and attendance on the important societies and clinics; to regard scrupulously the interests of my professional brothers, and seek their counsel when in doubt of my own judgment; to render willing help to my colleagues, and to give freely of my services to the needy. Moreover, I pledge myself, so far as I am able, to avoid the sins of selfishness, to shun unwarranted publicity, dishonest money-seeking and commercialism as disgraceful to our profession; to refuse utterly all secret money dealings with consultants and practitioners, to teach the patient his financial duty to the physician, and to urge the practitioner to obtain his reward from the patient openly; to make my fees commensurate with the service rendered and with the patients' rights, and to avoid discrediting my associates by taking unwarranted compensation. Finally, I pledge myself to co-operate in advancing and extending, by every lawful means in my power, the influence of the American College of Surgeons." A remarkable pledge, certainly, but one better suited, we think, to American than to British conditions, though there are many passages in it which surgeons in this country might well take to heart. We are too slow-moving in the Old World for bright ideas such as this scheme propounds to "catch on" as readily as they do in America; but we manage to "get there" in the end, somehow or other. We can scarcely imagine the English Royal College consenting to innovations such as these; but, then, it is not a democratic body. At the same time, there is a good deal of sound sense in the programme of the American Society, and no one can pretend that the present haphazard methods of education and training in surgery in this country are as satisfactory as they ought to be, and could be. On that point there will be many in agreement with Mr. Watson.

